# Allogeneic expanded adipose‐derived stem cells in the treatment of rectovaginal fistulas in Crohn’s disease

**DOI:** 10.1111/codi.15324

**Published:** 2020-09-04

**Authors:** M. Nikolic, A. Stift, W. Reinisch, H. Vogelsang, A. Matic, C. Müller, M. von Strauss und Torney, S. Riss

**Affiliations:** ^1^ Department of Surgery Medical University of Vienna Vienna Austria; ^2^ Department of Internal Medicine III Medical University of Vienna Vienna Austria; ^3^ Department of Visceral Surgery St Clara Hospital and University Hospital Basel Clarunis University Centre for Gastrointestinal and Liver Diseases Basel Switzerland

**Keywords:** Stem cells, Rectovaginal fistula, Crohn's disease

## Abstract

**Aim:**

Crohn's disease (CD)‐related rectovaginal fistulas (RVFs) are rare, challenging to treat and associated with a high morbidity. Due to a significant lack of data, we aimed to analyse the safety and feasibility of allogeneic adipose‐derived stem cells (ASCs) in the treatment of CD‐related RVF.

**Method:**

Four consecutive patients with CD‐related RVF underwent treatment with expanded allogeneic ASCs extracted from a healthy donor in a tertiary referral centre in 2019. None of the patients had an intestinal diversion at the time of the treatment. Follow‐up was performed 6 months postoperatively.

**Results:**

The median operation time was 45 min with a median hospital stay of 3 days. No intra‐operative complications occurred. Three patients (75%) developed recurrent RVF after a median follow‐up of 19 days. Two patients required surgical treatment including loose seton drainage due to discharge and pain. One patient developed recurrence of symptoms after 10 days, but refused further surgical therapy. Only one patient (25%) showed healing of the RVF, with re‐epithelialization of both the vaginal and rectal opening and absence of clinical symptoms.

**Conclusion:**

Expanded allogeneic ASC therapy represents a novel safe treatment option for CD‐associated RVF. Although efficacy appears limited, further controlled studies are required to draw robust conclusions.

**What does this paper add to the literature?**

Innovations in surgical therapy of rectovaginal fistulas (RVFs) lead to closure of the therapy gap between immunosuppressive and invasive treatment. This work shows that allogeneic adipose‐derived stem cells are a safe treatment option for Crohn's‐related RVFs. Thus, we hope to evoke interest and initiate further research to test treatment efficacy and draw stronger conclusions.

## Introduction

Rectovaginal fistula (RVF) is a rare but severe and particularly difficult disease to treat. RVFs represent around 5% of all anorectal fistulas [1, 2] and affect around 0.2–2.1% of patients with chronic inflammatory bowel disease, especially Crohn's disease (CD) [1, 3, 4]. The majority have symptoms ranging from passage of stool or gas from the vagina, resulting in urinary tract infections, dyspareunia, perineal pain and chronic vaginal discharge [3, 4, 5, 6]. In an effort to minimize symptoms and ultimately close the fistula women undergo multiple treatments, including systemic immunosuppressive medication as well as invasive surgery [2, 3, 4, 7, 8]. The treatment of RVF is challenging and associated with a significant risk of failure. Thus, approximately 40% of patients will choose to have a permanent colostomy for control of symptoms [4].

Nonetheless, a novel surgical approach to the treatment of RVFs has recently being studied in which adipose‐derived stem cells (ASCs) are injected around the fistula tract [4, 9, 10, 11, 12, 13]. The mode of action is based on the anti‐inflammatory and immune‐regulatory characteristics of stem cells which are supposed to accelerate healing of damaged tissue [14, 15]. The ASCs are extracted by liposuction from subcutaneous fat, and contain 100 times more stem cells than similar bone marrow aspirates [4, 9, 10, 11, 12, 13, 16]. Previous Phase I, II and III trials have shown safety as well as efficacy of autologous ASCs, not only in perianal fistulas in general, but also for fistulizing CD [9, 11, 16]. However, only a very limited number of these patients actually had RVFs due to CD [9, 11, 16]. Notably, one Phase I–II clinical trial examined allogeneic expanded ASCs from a healthy donor in the treatment of CD‐related RVF, revealing promising results [13]. Furthermore, a Phase III prospective, randomized, double‐blind, placebo‐controlled study also examined the use of allogeneic expanded ASCs (Alofisel®) in complex perineal fistulas in CD. [17] That study showed promising results, with a healing rate of 56.3% in patients receiving Alofisel® compared with 38.6% in the control group at 52 weeks, and encouraged us to investigate the same product in the treatment of RVF.

The aim of the present pilot study was to assess the safety and feasibility of allogeneic ASCs in the treatment of CD‐related RVF.

## Method

### Preoperative assessment

Four consecutive female patients with a median age of 52 years (range 32–66 years) received a standardized interview prior to treatment. The patients' histories were recorded and clinical examinations performed. Symptoms were scored using the Perianal Disease Activity Index (PDAI), and Crohn's Disease Activity Index (CDAI) [18, 19]. RVF was confirmed clinically and by routine pelvic MRI imaging. The median duration of RVF was 3 years (range 2–3 years). The only exclusion criterion was patients with active proctitis.

This study was approved by the Institutional Review Board of the Medical University of Vienna, Austria (ECS 1498/2018).

### Cell characteristics

The stem cell product named Alofisel® was obtained through Takeda Pharmaceutical Company Limited. Alofisel® contains a 5 million cells/ml suspension of expanded human allogeneic mesenchymal adult stem cells extracted from adipose tissue. A commercial unit contains four vials, each consisting of 30 million stem cells in 6 ml of solution, giving a total of 120 million stem cells in one commercial unit.

### Surgery

All procedures were performed under general anaesthesia by two specialized colorectal surgeons. After a single‐shot antibiotic dose of 1.5 g of ciprofloxacin and metronidazole an anal inspection was performed and the RVF was identified. Subsequently, the fistula was curetted thoroughly and irrigated with saline. A vaginal and rectal mucosal flap was created to achieve a tension‐free closure of the internal opening using 3.0 absorbable sutures. Finally, one vial of stem cell solution (6 ml) was injected around the entire short fistula tract and the vaginal and rectal opening was closed.

Oral antibiotics (ciprofloxacin and metronidazole or amoxicillin and clavulanic acid) were continued for 5 days postoperatively.

### Postoperative assessment

Patients were followed up until 6 months after surgery. Postoperative complications and drug‐associated adverse events were recorded. In addition, short‐term healing rates, defined by absence of vaginal secretion and closure of vaginal and rectal fistula opening on clinical exam, were evaluated.

### Statistical analysis

Because of the limited number of patients we did not perform a statistical analysis.

## Results

### Demographic data

Preoperative patient characteristics are outlined in Table 1. None of the patients had active proctitis. In all participants, immunosuppressive and/or biological therapy failed to heal the RVF. While patient 1 had only received medical treatment the remaining patients had been operated on for RVF prior to stem cell therapy. None of the four patients had an intestinal diversion at the time of the ASC treatment.

**Table 1:** Demographics of patients operated on for rectovaginal fistula (RVF).

Patient ID	Age (years)	Duration of CD (years)	Duration of RVF (years)	No. of tracts	No. of previous surgeries*	Previous medication	Ongoing medication	PDAI	CDAI	Reoccurrence (days)
1	56	25	2	1	0	Corticosteroids	Adalimumab, mesalamine	6	196	–
2	66	40	2	1	> 3	Prednisolone	Ayurvedic medication	15	208	19
3	32	11	3	1	> 3	Corticosteroids, infliximab, adalimumab, azathioprine, mesalamine, ustekinumab	Azathioprine, mesalamine	10	177	10
4	48	11	3	1	2	Azathioprine, mesalamine, sulfasalazine, infliximab, prednisolone	Azathioprine, mesalamine, sulfasalazine, infliximab	5	81	42

CD, Crohn’s disease; CDAI, Crohn's Disease Activity Index; PDAI, Perianal Disease Activity Index.

* Crohn's disease and RVF‐related surgeries.

Patient 2 had a history of several intestinal and colonic resections for CD. A loose seton drainage was inserted into the RVF 2 months prior to stem cell therapy.

Patient 3 received an ileocaecal resection due to CD. Before the treatment of the RVF she underwent placement of a loose seton drainage, followed by a fistula extraction and vaginal flap placement. Five months later the recurrent RVF was treated again with a mucosal rectal flap combined with an intestinal diversion. Finally, the patient underwent an intestinal reconstruction 5 months later when the fistula was considered to be closed. Two years later the RVF recurred.

Patient 4 underwent an ileocaecal resection and a resection of the sigmoid colon due to an entero‐sigmoidal fistula. The RVF was treated with an insertion of a loose seton drainage 3 years prior to the stem cell therapy.

The preoperative median PDAI was 5.5 (5–15), while the CDAI was 138.5 (81–208).

### Surgical data

The median operation time time was 45 min (range 20–60 min). All surgical procedures had an uneventful intra‐operative course.

### Postoperative outcome

Only patient 1 (*n* = 1, 25%) achieved a closure of the RVF, assessed by a physician with a clinical rectal and vaginal exam, and full symptom relief. She had her first follow‐up 10 days after the surgery showing healing of the RVF. At 6 months' follow‐up the RVF remained closed.

The remaining patients (*n* = 3, 75%) developed early recurrence of the RVF‐associated symptoms and showed open RVF on clinical exam after a median follow‐up of 19 days. Two (*n* = 2, 50%) patients underwent further surgical interventions.

Patient 2 presented to the emergency department with a recurrence of faecal discharge and perineal pain 19 days after the stem cell injection. On clinical examination the RVF was open. She underwent emergency surgery including the placement of a loose seton drainage. Four months later the patient received a terminal colostomy followed by an interposition of a Gracilis flap 2 months later. At the 4 week follow‐up, the patient reported reduction of pain and minimal clear secretion.

Patient 3 developed recurrence of vaginal drainage 10 days following surgery. The RVF was also open on clinical exam, but did not require emergency surgery. However, 7 months later an ileostomy was constructed and a Gracilis flap procedure is scheduled for the near future.

Patient 4 was readmitted due to fever 42 days after the stem cell intervention. A loose seton was re‐inserted to drain the opening of the RVF. In addition, the patient developed bacteraemia with Gram‐positive streptococci. After adapted antibiotic therapy the bacteraemia was successfully treated and the patient was discharged.

## Discussion and conclusion

CD‐related RVFs develop as a result of local inflammation, and affect up to 2.1% of all patients [1, 2, 3, 4, 6, 7, 8, 20, 21]. The quality of life of these patients is significantly decreased due to the wide range of symptoms caused by RVF, from perineal discomfort to passage of stool through the vagina [1, 2, 4, 6, 7, 11, 21]. Depending on the size, location, associated rectal involvement and patient characteristics a variety of medical and surgical treatments exist [8]. When systemic immunosuppressive treatment fails to ease symptoms or close the fistula tract, surgical therapy with anorectal flaps or interposition grafts is next possible approach [2, 6, 20, 22, 23]. Nonetheless, the success rates for primary closure and advancement flaps still vary from 60% to 100%, leaving up to half of patients unsatisfied, with a diminished quality of life [2, 7]. Hence, novel therapies such as ASCs are of increasing importance.

The use of stem cells in the treatment of RVF was encouraged by results of Phase I, II and III studies conducted in Europe and Israel [9, 11, 16]. It is hypothesized that the immunoregulatory and anti‐inflammatory qualities of stem cells accelerate healing of inflamed and damaged tissue [14, 15]. Consequently, Garcia‐Olmo *et al*. used stem cells in the treatment of CD‐related perianal fistulas in four patients. That study showed promising results, with a healing rate of up to 75% with no risk of incontinence and no observed side effects, so stem cell therapy became of increasing interest for perianal CD [16]. By extracting the stem cells from adipose tissue rather than bone marrow, the number of stem cells is significantly increased and the rate of adverse effects is reduced [4, 9, 10, 11, 12, 16]. Additionally, allogeneic ASCs retrieved from a healthy donor can also be used, further reducing the inherent costs and time‐consuming processing of autologous ASCs [13].

To date, data on ASCs in the treatment of RVF in patients with CD are scarce. Thus, in this study we aimed to analyse the safety and feasibility of allogeneic ASCs in the treatment of four consecutive CD‐associated RVFs. After the fistula was curetted thoroughly and irrigated with saline, a vaginal and rectal mucosal flap was created to close the internal orifices and a 6 ml solution of Alofisel® containing 30 million stem cells was finally injected into the RVF tract. The creation of a rectal or vaginal flap before injecting the ASCs was encouraged by previous studies using ASCs in the treatment of perineal and rectovaginal fistulas, as well as in another study showing a success rate of up to 81% in healing of RVFs and very low morbidity [12, 13, 24].

At 6 months' follow‐up, we observed healing of the RVF in one out of four patients (*n* = 1/4, 25%). The patient who showed complete healing was the only patient who had no previous operations due to CD, thus the one with less severe disease. This finding shows that disease severity could have an effect on the efficacy of the treatment and healing of the RVF. Furthermore, ASCs could be more effective as first‐line treatment for RVF rather than after multiple procedures and recurrences.

Our results are worse than those previously described in two studies showing complete healing in 60% and 75% of patients, respectively [11, 13]. As individual medical history and CD severity vary among patients, accurate comparison is often difficult. Furthermore, different applications may influence outcome. Our patients received only a single dose of 30 million ASCs, instead of a primary dose of 20 million ASCs followed by an additional dose of 40 million ASCs if no healing was observed at 8 or 12 weeks' follow‐up or a higher one‐time dose of 120 million ASCs [11, 13, 17]. Due to the anatomy of RVFs and the thin fibrous tissue between the rectum and vagina, most commonly no more than 6 ml of solution can be injected into the tract. In contrast, complex perineal fistulas require a larger number of cells due to longer tracts and their different locations. Thus, each patient received an initial 6 ml suspension with 30 million ASCs, higher than in the above‐mentioned studies. Unfortunately, as the RVF persisted/recurred and three of the patients developed recurrence of symptoms, the tissue surrounding the RVF was inflamed and an additional dose of ASCs at that time was not possible. To date, no protocol exists concerning the ideal dose of ASCs in the treatment of RVF. This explains the large discrepancies in dose application, which may also affect closure rates. Another difference that could explain the lower healing rate in our study was that Garcia‐Olmo *et al*. separately evaluated CD‐related fistulas and RVFs, not further specifying the origin of the RVF. Thus, it remains unknown whether some of those may have been CD related. This could be of importance as concomitant inflammatory bowel disease could be an explanation for reduced or delayed healing of CD‐related fistulas in comparison with fistulas with different aetiologies [11]. Moreover, Garcia‐Arranz *et al*. excluded patients with CDAI > 201 and administration of anti‐tumour necrosis factor during the previous 8 weeks [13]. We had two patients who fulfilled each of these criteria (Table 1).

Herreros *et al*. [12] conducted a compassionate use programme for stem cells in complex perianal fistulas. In total, seven patients had RVFs, only three of which were CD related. They reported less successful results, with no healing at 6 months' follow‐up and six patients showing improvement, defined as closure of 50% of external orifices, vaginal and rectal, or 50% decrease of suppuration from the external orifices. The included patients received a reduced dose of 20 million ASCs, although only one RVF patient from seven was treated with ASCs. The other six patients were treated with the stromal vascular fraction from a lipoaspirate.

Most recently, Lightner *et al*. [4] evaluated the implantation of an autologous adipose‐derived MSC‐coated fistula plug in five CD patients with RVF. They reported no case of complete clinical remission, defined as complete cessation of drainage and lack of rectovaginal tract under anaesthesia, and radiographic remission, defined as no visible rectovaginal tract on MRI, at 6 months' follow‐up. Nevertheless, three patients (60%) described clinical healing with total cessation of drainage. Besides the difference in the application of ACSs another possible explanation for a better clinical outcome compared with our study is that all patients had an intestinal diversion at the time of surgery. Certainly, a stoma could potentially help to increase the healing rate. Although we discussed this option and the potential benefits with the patients, they showed a great reluctance to have an ostomy before stem cell injection. As the stem cell injection is considered a minimally invasive approach, we decided to assess the effectiveness of ASCs in patients without intestinal diversion.

We observed no intra‐operative or directly postoperative complications or adverse effects, such as bleeding, infection, allergic reactions or sepsis. This is in accordance with previous trials, verifying the safety of the treatment in CD patients with RVF [4, 11, 12, 13]. Nevertheless, two patients required an emergency operation with drainage of the abscess and subsequent antibiotic therapy caused by persistence of the RVF. Late complications, such as perineal abscess or even sepsis due to treatment failure, should not be underestimated.

A few limitations of our study need to be addressed. Although the number of included patients was low, we still believe our results are of clinical importance. Taking into account the significant lack of data in the literature with regard to stem cell therapy for RVF, our results can provide further evidence for this particular subset of patients. In addition, healing was defined as re‐epithelialization of both the vaginal and rectal opening, assessed by a physician, and total absence of clinical symptoms. Thus, no radiographic imaging was done to prove complete closure of the fistula tract.

The diverse results, as well as the diversity of the few conducted studies, are further proof of the complexity of RVFs and their treatment, but also of the rarity of the disease. Allogeneic as well as autologous ASCs represent a potential, safe and novel treatment, lacking all the adverse effects associated with medical and existing surgical therapies.

## Conclusion

Expanded allogeneic ASC therapy represents a novel treatment option for CD‐associated RVF. Although the treatment appears to be safe and feasible, the healing of the fistula tract was achieved in only one out of four patients without concomitant intestinal diversion. Further randomized prospective studies are required to draw final conclusions.

## Funding

No financial support was received for this study.

## Conflicts of interest

AS, SR, WR and HV received fees/honoraria (e.g. lectures, advisory boards) from Takeda. MN, CM, AM and MvSuT have no conflicts of interest to declare.

## Ethical approval

This study was approved by the Institutional Review Board of the Medical University of Vienna, Austria (ECS 1498/2018).

## Author contributions

All authors listed above contributed substantially to the conception or design of the work and the acquisition, analysis or interpretation of data for the work; and all authors contributed to the drafting of the work or revising it critically for important intellectual content and the final approval of the version to be published. All authors agree to be accountable for all aspects of the work in ensuring that questions related to the accuracy or integrity of any part of the work are appropriately investigated and resolved.

## Data Availability

The data that support the findings of this study are available on request from the corresponding author. The data are not publicly available due to privacy or ethical restrictions.
